# Estimating the causal effect of frailty index on vestibular disorders: A two-sample Mendelian randomization

**DOI:** 10.3389/fnins.2022.990682

**Published:** 2022-08-24

**Authors:** Gui Xiao, Hu Wang, Jiaji Hu, Li Liu, Tingting Zhang, Mengjia Zhou, Xingxing Li, Chunxiang Qin

**Affiliations:** ^1^Department of Health Management, The Third Xiangya Hospital, Central South University, Changsha, China; ^2^Xiangya School of Nursing, Central South University, Changsha, China

**Keywords:** frailty index, vestibular disorders, Mendelian randomization, dizziness, vertigo

## Abstract

**Background:**

Frailty index and vestibular disorders appear to be associated in observational studies, but causality of the association remains unclear.

**Methods:**

A two-sample Mendelian randomization (MR) study was implemented to explore the causal relationship between the frailty index and vestibular disorders in individuals of European descent. A genome-wide association study (GWAS) of frailty index was used as the exposure (*n* = 175, 226), whereas the GWAS of vestibular disorders was the outcome (*n* = 462,933). MR Steiger filtering method was conducted to investigate the causal effect of the frailty index on vestibular disorders. An inverse variance weighted (IVW) approach was used as the essential approach to examine the causality. Additionally, the MR-Egger methods, the simple mode analysis, the weighted median analysis, and the weighted mode analysis were used as supplementary methods. The MR-PRESSO analysis, the MR-Egger intercept analysis, and Cochran's Q statistical analysis also were used to detect the possible heterogeneity as well as directional pleiotropy. To evaluate this association, the odds ratio (OR) with 95% confidence intervals (CIs) was used. All statistical analyses were performed in R. The STROBE-MR checklist for the reporting of MR studies was used in this study.

**Results:**

In total, 14 single nucleotide polymorphisms (SNPs) were identified as effective instrumental variables (IVs) in the two sample MR analyses. The significant causal effect of the frailty index on vestibular disorders was demonstrated by IVW method [OR 1.008 (95% CI 1.003, 1.013), *p* = 0.001]. Results from the various sensitivity analysis were consistent. The “leave-one-out” analysis indicated that our results were robust even without a single SNP. According to the MR-Egger intercept test [intercept = −0.000151, SE = 0.011, *p* = 0.544], genetic pleiotropy did not affect the results. No heterogeneity was detected by Cochran's Q test. Results of MR Steiger directionality test indicated the accuracy of our estimate of the potential causal direction (Steiger *p* < 0.001).

**Conclusion:**

The MR study suggested that genetically predicted frailty index may be associated with an increased risk of vestibular disorders. Notably, considering the limitations of this study, the causal effects between frailty index and vestibular disorders need further investigation. These results support the importance of effectively managing frailty which may minimize vestibular disorders and improve the quality of life for those with vestibular disorders.

## Introduction

The vestibular disorders (VDs) include a variety of syndromes or (and) diseases arising from disfunction of the inner ear, vestibulocochlear nerve, or central vestibule (Strupp et al., 2020). The symptoms of vestibular disorders involve vertigo, dizziness, and imbalance, which have a strong impact on the daily life and health (Bisdorff et al., 2015). There is a high incidence of vestibular disorders. According to data from the 2001–2004 National Health and Nutrition Examination Surveys (*n* = 5,086), 35.4% of US adults aged 40 years and older had vestibular dysfunction (Agrawal et al., 2009). The prevalence of dizziness was 16.70% in South Korea according to data from the 2009 to 2010 Korea National Health and Nutrition Examination Surveys, which were cross-sectional surveys of the South Korean civilian, non-institutionalized population aged 40 years and older (*n* = 3,267) (Koo et al., 2015). Studies have shown that dizziness and vertigo are highly prevalent in the community over the past decade. Dizziness (including vertigo) affects about 15% to over 20% of adults yearly in large population-based studies (Neuhauser, 2016). It is estimated that vestibular disorders pose a substantial burden on the healthcare system because of the high prevalence and severity of symptoms (Kobel et al., 2021). Moreover, vestibular disorders are among the most relevant contributors to the burden of disability among older adults living in the community and associated with immobility, limitations of activities of daily living and decreased participation (Regauer et al., 2020). Studies have focused on factors or diseases associated with vestibular disorders, such as gender, age, hyperglycemia and hypertension, thyroid function abnormalities, abnormal lipid metabolism, and abnormal sex hormone levels (Grill et al., 2016; Bronstein and Dieterich, 2019; Brandt and Dieterich, 2020). Vestibular disorders have been associated with these factors or diseases, but the causal link has not been fully established. Understanding the potential association between vestibular disorders and related diseases or factors, and the underlying mechanisms may facilitate the individualized management and early interventions of patients with vestibular disorders.

Frailty refers to a complex clinical syndrome with the characteristics of a decrease in physiological capacity across multiple organs or systems, as well as an increase in susceptibility to stress (Dent et al., 2019). Frailty increases the risk of hospitalizations, iatrogenic complications, falls and fractures, lower quality of life, and early mortality and other health problems in older people (Vermeiren et al., 2016; Cesari et al., 2017; Junius-Walker et al., 2018; Yang et al., 2018). Frailty index is widely accepted as one operationalization of frailty (Martin and O'Halloran, 2020). The frailty index is a continuous measurement based on the rate of the number of health defects due to aging to all the number of defects considered (Kojima et al., 2018; Palliyaguru et al., 2019). Defects can be manifested as symptoms, signs, diseases, disabilities, laboratory abnormalities, radiographic abnormalities, and social characteristics. The frailty index measurements take into account diverse aspects of health contemporaneously and are highly predictive of a variety of pernicious consequences, such as functional degenerating, physical disabilities, falls, death and morbidity (Theou et al., 2016; Bersani et al., 2020). Thus, the frailty index is a highly sensitive method for representing frailty. Evidence from numerous epidemiological research proved that frailty increases the dizziness risk (de Moraes et al., 2011, 2013; Gomez et al., 2011; Kammerlind et al., 2016). However, the relationship remains doubtful as a result of contrary causality and confounding factors. A randomized controlled trial (RCT) is regarded as the reliable method of demonstrating the causal relationship between frailty index and vestibular disorders (Eichler et al., 2021). Regrettably, it is not usually feasible to conduct RCT because of the complexities of methodology, financial restrictions, and/or difficulty of collecting sufficient samples (Evans and Davey Smith, 2015; Gupta et al., 2017).

Mendelian randomization (MR) analysis is a useful epidemiological research strategy for assessing causal relationships. In MR, genetic variants are used as instrumental variables (IVs) for assessing the causal effect between exposure and outcome. With MR, genotypes can be unbiasedly estimated as they are determined at conception and they are generally not confounded by other factors such as reverse causation (Bowden and Holmes, 2019). Due to this huge advantage, MR has been widely applied in recent years to infer causality from publicly available GWAS summary statistics (Hartwig et al., 2017; Choi et al., 2020; Wang et al., 2022). Herein, a two-sample MR approach was implemented to assess the potential causal effect of frailty index on vestibular disorders in the study.

## Materials and methods

### MR design and data source

**Figure 1:**
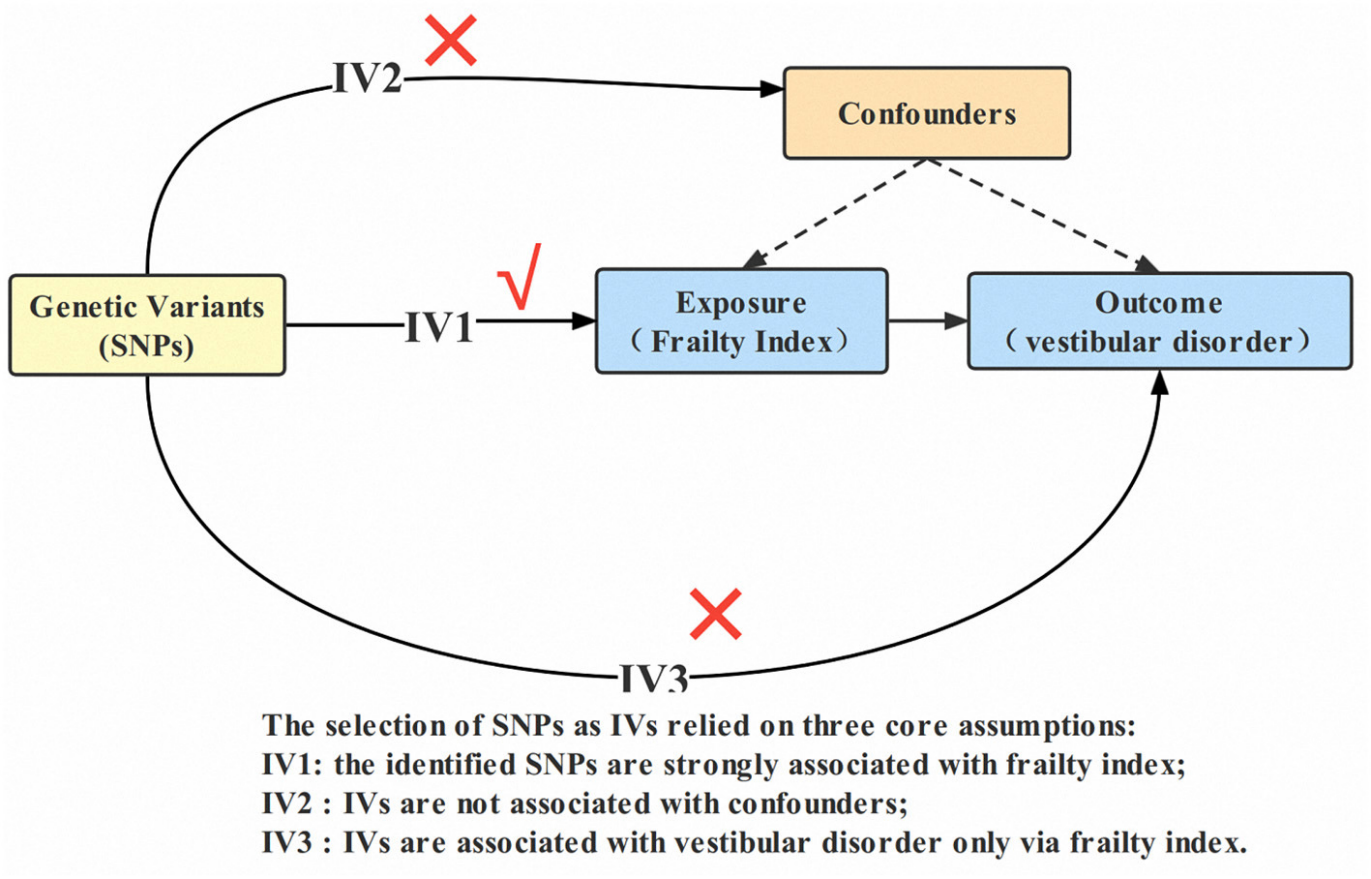
Design of the two-sample Mendelian randomization study. IVs, instrumental variables; SNP, single nucleotide polymorphism.

The general design of this MR research can be found in Figure 1. The study methods were compliant with the STROBE-MR checklist (Skrivankova et al., 2021). The complete GWAS summary statistics for the frailty index can be downloaded from the GWAS catalog checklist. (https://www.ebi.ac.uk/gwas/downloads/summary-statistics; study accession GCST90020053). A detailed description of the exposures and outcomes of the GWAS used in the MR study can be found in Supplementary Table 1.

### Frailty index GWAS dataset

The GWAS involving 175, 226 European ancestry individuals were used to generate the exposure dataset for frailty index. (https://gwas.mrcieu.ac.uk/datasets/ebi-a-GCST90020053/). Notably, Atkins et al. (2021) reported the most comprehensive exploration of genetic influences on the frailty index so far, by performing a genome-wide association study (GWAS) meta-analysis of the frailty index data in European descent UK Biobank participants (*n* = 164,610, 60–70 years old) and Swedish Twin Gene participants (*n* = 10,616, 41–87 years old). For UK Biobank and Twin Gene, frailty indexes were calculated using 49 or 44 self-reported items on symptoms, disabilities, and diagnosed diseases. The 49 self-reported baseline data variables were used to calculate the frailty index for UK Biobank. Physiological and mental health variables, including symptoms, disabilities, and diagnosed diseases, were reported by participants at baseline (see Supplementary Table 2 for details of the frailty index components included). The 44 deficits were used to calculate the TwinGene's frailty index (see Supplementary Table 3 for details of the frailty index components included). Of the 49 items used in UK Biobank, 29 of these have approximate items in TwinGene. In total, 14 loci were related with the frailty index (*p* < 5^*^10^−8^).

### Vestibular disorders GWAS dataset

In terms of the GWAS outcome datasets, vestibular disorders were taken from a different independent study that included 462,933 individuals (4,012 cases and 458,921 controls) of European ancestry (https://gwas.mrcieu.ac.uk/datasets/ukb-b-5188/). The GWAS summary data for vestibular disorders was obtained from the MRC-IEU Open GWAS data infrastructure, available through the UK Biobank (Elsworth et al., 2020). UK Biobank is a large, population-based prospective cohort study that enables health-related research. It has already been described in detail how the study will be designed and who will be participating in it. Over 500,000 participants were recruited between 2006 and 2010 for the UK Biobank. The participants provided detailed data *via* questionnaires and verbal interviews, as well as phenotypic data and biological samples. The assessment of vestibular disorders visit comprised electronic signed consent; a self-completed touch-screen questionnaire; brief computer-assisted interview (Sudlow et al., 2015).

### Selection of instrumental variables (IVs)

There are three assumptions that must be satisfied by the IVs used in MR analysis: (1) IVs must be relevant to exposure (i.e., frailty index); (2) IVs must be independent of any confounding factor; and (3) IVs are associated with outcome (i.e., vestibular disorders) only *via* exposure (i.e., frailty index) (Burgess et al., 2013). As a first step, the independent genetic variants (SNPs) with significant genome-wide associations (*p* < 5 × 10^−8^) for frailty index were identified as IVs. Then, independent variants were identified using a clumping procedure implemented in R software, in which a linkage-disequilibrium threshold of r^2^ < 0.001 within a 10,000 kb window in the European 1,000 Genomes Project Phase 3 reference panel was set (Machiela and Chanock, 2015; Myers et al., 2020). The LD of chosen SNPs strongly related to frailty should meet some criteria, for example, r^2^ < 0.001 and distance >10,000 kb (Myers et al., 2020). From the chosen instrumental SNPs, palindromic SNPs with middle allele frequency were removed (A palindromic SNP is a SNP with the A/T or G/C allele, whereas the “middle allele frequency” are from 0.01 to 0.30). MR Steiger filters are used to exclude SNPs with the incorrect causal direction. Due to their low confidence level, SNPs with a minor allele frequency < 0.01 were also eliminated from the original GWAS. Finally, we calculated the explained variance (R^2^) and F statistic parameters to determine whether the identified IVs were powerful enough. Generally, IVs (SNPs) with F-statistic parameters <10 are considered weak instruments (Burgess et al., 2017).

### MR analyses

Wald ratios were computed to calculate the causal impact of exposure on site-specific outcome mediated by instrumental SNPs (Yu et al., 2015). To calculate the strength of the association between frailty index and vestibular disorders, inverse variance weighted (IVW) approach was used as the essential analysis method in our study. In addition, MR-Egger method, weighted median method, and simple mode method were conducted as supplementary methods (Qi and Chatterjee, 2019). Odds ratios (OR) were used to measure causal effects. in addition, it was calculated using Cochran's Q statistic in order to estimate the heterogeneity of each SNP (Cohen et al., 2015; Wang, 2022). In order to assess the bias caused by ineffective IVs and the possibility of horizontal pleiotropy, MR-Egger intercepts and MR-PRESSO were used (Bowden et al., 2015; Burgess and Thompson, 2017; Verbanck et al., 2018). A “leave-one-out” sensitivity analysis was also conducted in order to determine if a single SNP affected the results (Burgess and Thompson, 2017). Moreover, we used the MR Steiger directionality test to examine whether the results we found followed the direction in our hypothesis. To adjust for confounders, a multivariable MR analysis was performed after MR analysis (Burgess and Thompson, 2015). In this multivariable MR analysis, genetic variants associated with at least one exposure were included, and a multivariable IVW procedure was used to estimate the causal relationship (Burgess and Thompson, 2015). Atkins et al. demonstrated that multiple traits are associated with the risk of frailty, including body mass index (BMI), C-reactive protein (CRP), inflammatory bowel disease (IBD), and smoking initiation (Atkins et al., 2021; Liu et al., 2022). As a result, we included the four covariates in the following multivariable analysis. BMI genetic variants were obtained from the GIANT consortium (Locke et al., 2015). The genetic variants for CRP were obtained from Wojcik et al. (2019). Genetic variants for IBD were obtained from the IIBDGC consortium (Liu et al., 2015). Genetic variants for smoking were obtained from the GSCAN consortium (Liu et al., 2019). Notably, all analyses were conducted using Two Sample MR 0.5.6 and MR-PRESSO packages in R (version 4.2.0, the R Foundation for Statistical Computing, Vienna, Austria). Statistical significance was determined by *p* < 0.05 with two-tailed tests. An FDR correction based on Benjamini-Hochberg was implemented to correct for multiple comparisons (Reiss et al., 2012).

## Results

### Strength of the instrumental variables

A two-sample MR analysis was applied to examine the causal association between frailty index and vestibular disorders. The generated IVs including 14 SNPs could explain 0.318% of the variance of their corresponding frailty index. In addition, the minimum F statistic of these IVs was 30, suggesting that all IVs were sufficiently effective for the MR analysis (F statistic >10). There were 14 SNPs involved in our analyses, as shown in Table 1.

**Table 1:** The characteristics of 14 SNPs and their genetic associations with frailty index and vestibular disorders.

**SNP**	**Chr**	**EA**	**OA**	**EAF**	***F*-statistics**	**SNP-frailty index**			**SNP-vestibular disorders**		
						**association**			**association**		
						**Beta**	**SE**	***p-*value**	**Beta**	**SE**	***p-*value**
rs10891490	11	C	T	0.5915	31	−0.0188	0.0034	2.00E-08	−0.000407302	0.000196225	0.03
rs12739243	1	C	T	0.2206	37	−0.0242	0.004	1.28E-09	0.000339685	0.000233882	0.15
rs1363103	5	C	T	0.38	32	−0.0191	0.0034	2.23E-08	2.02E-05	0.000199038	0.92
rs17612102	15	C	T	0.5933	30	0.0187	0.0034	2.85E-08	6.23E-05	0.000196554	0.75
rs2071207	3	C	T	0.478	32	−0.0187	0.0033	1.47E-08	−0.000154846	0.000192738	0.42
rs2396766	7	A	G	0.4725	37	0.0201	0.0033	1.22E-09	0.000251141	0.000193096	0.19
rs3959554	15	G	A	0.4177	31	0.0189	0.0034	1.74E-08	0.00015428	0.000195434	0.43
rs4146140	10	T	C	0.3811	34	−0.0198	0.0034	6.83E-09	−2.96E-05	0.000199535	0.88
rs4952693	2	T	C	0.3734	33	−0.0194	0.0034	1.47E-08	−3.85E-05	0.000199655	0.85
rs56299474	8	A	C	0.1733	30	0.0241	0.0044	3.94E-08	−4.98E-05	0.00025414	0.84
rs583514	3	C	T	0.5111	36	0.0199	0.0033	1.65E-09	0.000195733	0.000192951	0.31
rs8089807	18	T	C	0.1866	33	−0.0248	0.0043	6.50E-09	−0.000436891	0.000248114	0.07
rs82334	4	C	A	0.3177	41	−0.0223	0.0035	3.13E-10	−0.000355566	0.000207515	0.08
rs9275160	6	A	G	0.3397	119	0.0382	0.0035	7.18E-28	0.000475587	0.00020293	0.01

*SNP, single nucleotide polymorphism; Chr, chromosome; EA, effect allele; OA, other allele; EAF, frequency of effect allele; SE, standard error*.

### MR and sensitivity analyses

**Figure 2:**
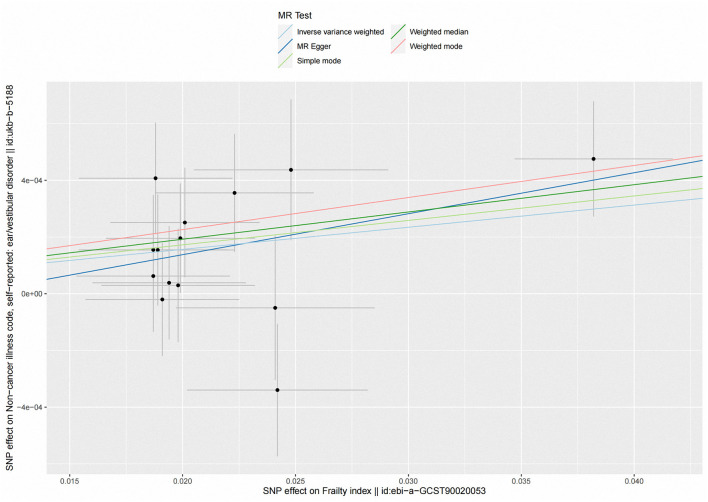
Scatter plot of the effects of genetic variants on frailty index and vestibular disorders. The slopes of the solid lines denote the magnitudes of the associations estimated from the MR analyses. MR, Mendelian randomization; SNP, single-nucleotide polymorphism.

**Figure 3:**
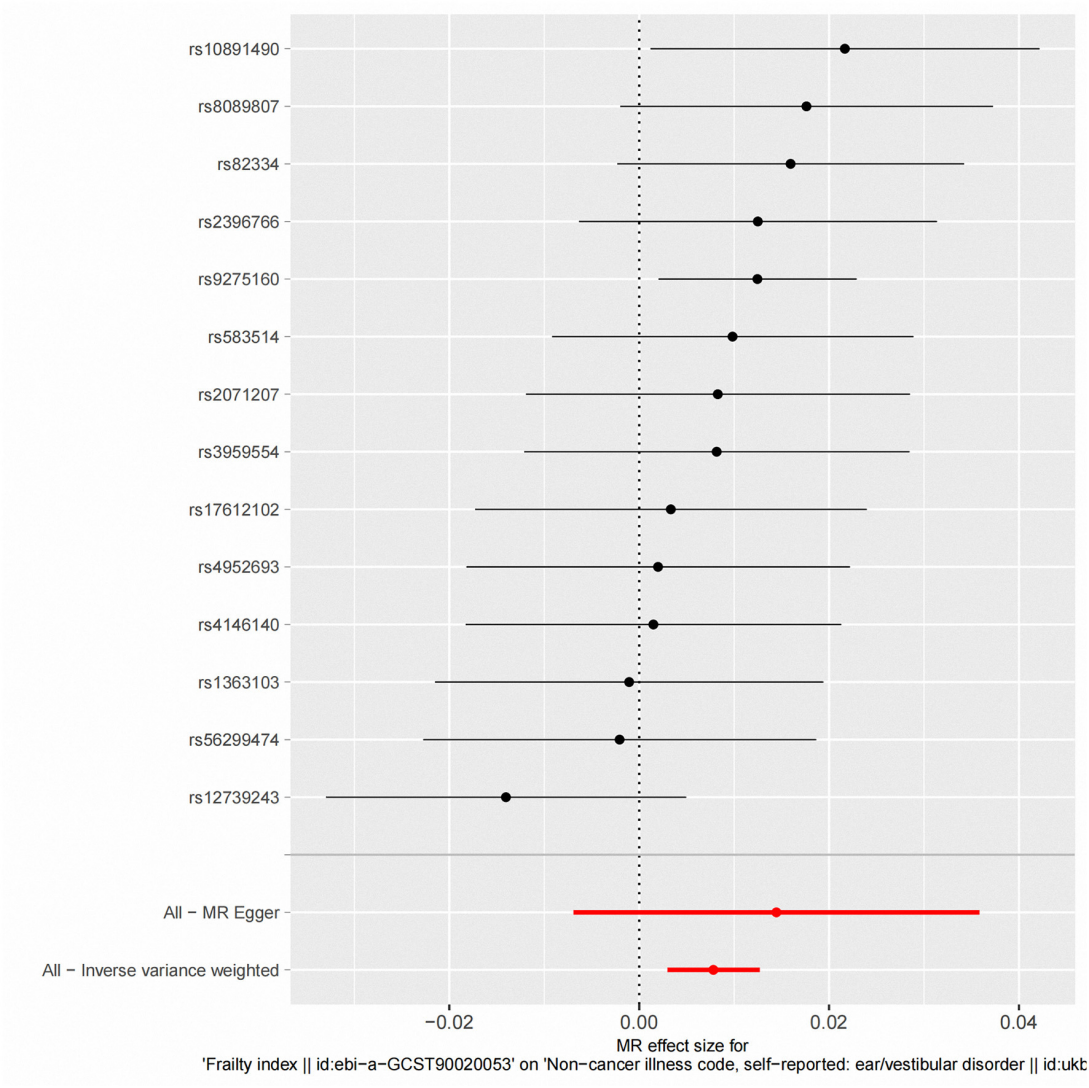
Fixed-effect IVW analysis of the causal association between frailty index and vestibular disorders. The black dots and bars indicate the causal estimate and 95% CI using each SNP. The red dot and bar indicate the overall estimate and 95% CI meta-analyzed by fixed-effect IVW method. IVW, inverse-variance weighted; HI, hearing impairment; CI, confidence interval; SNP, single nucleotide polymorphism.

**Figure 4:**
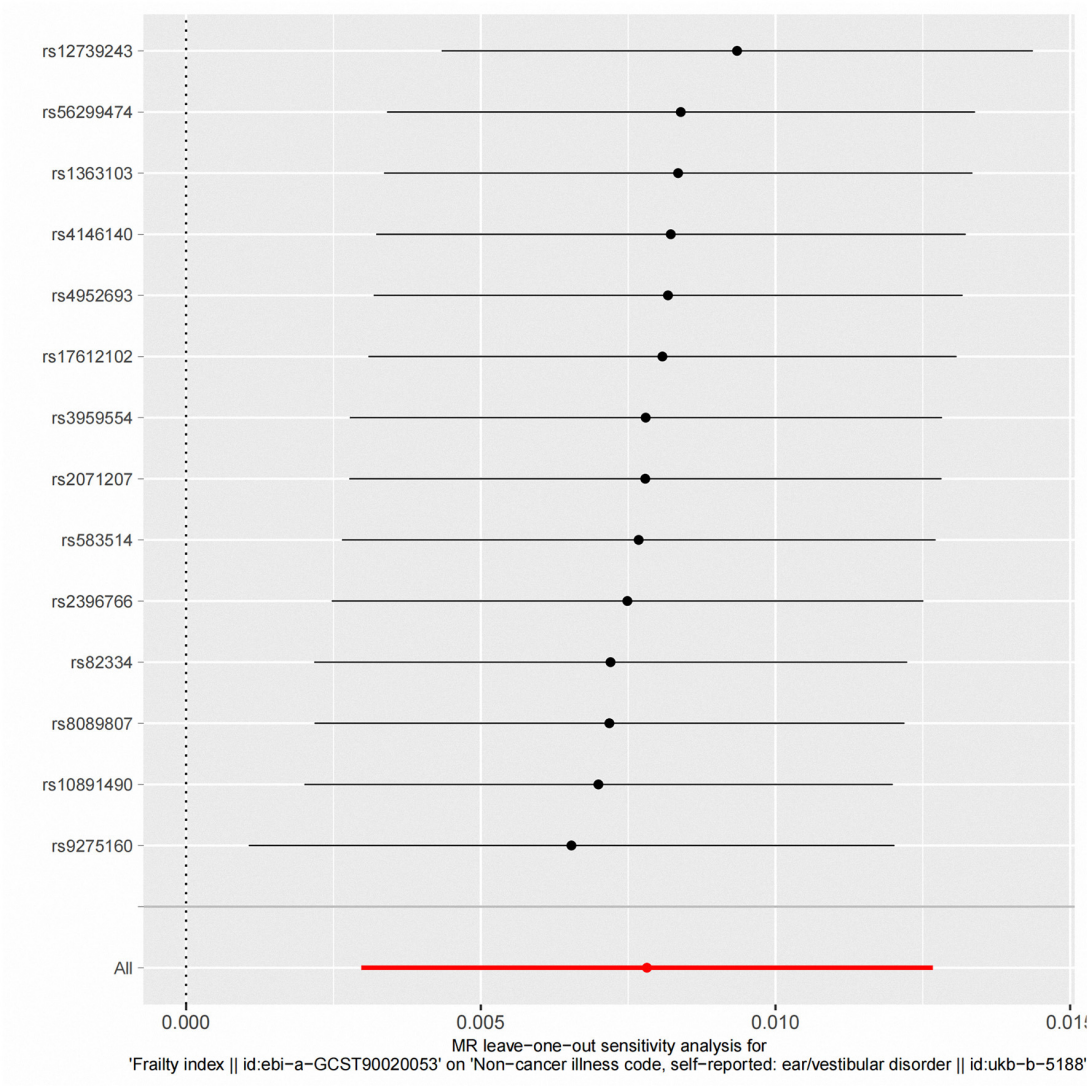
“Leave-one-out” analysis of the causal association of frailty Index and vestibular disorders. The black dots and bars indicate the causal estimate and 95% CI when an SNP was removed in turn. The red dot and bar indicate the overall estimate and 95% CI using the fixed-effect IVW method; CI, confidence interval; SNP, single nucleotide polymorphism; IVW, inverse-variance weighted.

According to fixed-effect IVW estimates, frailty index significantly contributed to an increased risk of vestibular disorders [OR 1.008 (95% CI 1.003, 1.013), *p* = 0.001] (shown in Table 2; Figures 2, 3). For the IVW method, Cochran's Q statistic was 11.75 (*p* = 0.466), suggesting a low level of heterogeneity and relative reliability of the causal effect. Additionally, weighted median analysis [OR 1.010 (95% CI 1.003, 1.017), *p* = 0.005], simple mode analysis [OR 1.009 (95% CI 0.998, 1.020), *p* = 0.162], weighted mode analysis [OR 1.011 (95% CI 1.002, 1.021), *p* = 0.036], and MR-Egger analysis [OR 1.015 (95% CI 0.993, 1.036), *p* = 0.209] also indicated comparable results (Table 2; Figure 2). MR analysis turned out to be reliable according to the results based on the “leave-one-out” analysis (shown in Figure 4; Supplementary Table 4). The horizontal pleiotropy between IVs and outcomes was investigated using MR-Egger regression, but no significant intercept was found [intercept = −0.000151, SE = 0.011, *p* = 0.544] (shown in Table 3). Furthermore, MR-PRESSO results indicated that horizontal pleiotropy did not exist in the MR study (*p* = 0.491). Based on the funnel plot results (shown in Supplementary Figure 1), there was neither horizontal pleiotropy nor heterogeneity in our MR study. Results of the MR Steiger directionality test indicated the accuracy of our estimate of the causal direction (Steiger *p* < 0.001). The estimated causal effect of frailty index on vestibular disorders may be still significant after adjustment for BMI (OR = 1.006, 95% CI 1.000–1.012, FDR-corrected *p* = 0.035), CRP (OR = 1.008, 95% CI 1.003–1.012, FDR-corrected *p* = 0.0007), IBD (OR = 1.006, 95% CI 1.000–1.012, FDR-corrected *p* = 0.035) and smoking (OR = 1.008, 95% CI 1.004–1.012, FDR-corrected *p* = 0.0007) (shown in Supplementary Tables 5, 6). As a consequence, we found that the frailty index may be causally related to vestibular disorders.

**Table 2:** Association of frailty index with vestibular disorders using various methods.

**Methods**	**OR**	**LCI**	**UCI**	***p*-value**
Fixed-effect IVW	1.00784921	1.00297777	1.01274430	0.001**
Weighted median	1.00965905	1.00297798	1.01638462	0.005**
Simple mode	1.00865773	0.99721538	1.02023138	0.162
Weighted mode	1.01137620	1.00185004	1.02099293	0.030*
MR-Egger	1.01455225	0.99311915	1.03644791	0.209

**p < 0.01; *p < 0.05; OR, odds ratio; LCI, lower confidence interval; UCI, upper; IVW, inverse-variance weighted.

**Table 3:** MR-Egger intercept test results of the association between frailty index and vestibular disorders.

**Estimates**	**SE**	**LCI**	**UCI**	***p*-value**
−0.000151	0.011	−0.007	0.036	0.544

## Discussion

Based on the summary level data from large GWAS, we implemented the two-sample MR study with the purpose of investigating the causal association between frailty index and vestibular disorders. We found a causality between genetically predicted frailty index and vestibular disorders in our analysis. The sensitivity analyses showed consistent estimates, indicating that there was minimal horizontal pleiotropy and the association was robust. According to our knowledge, this is the first MR study to evaluate the potential causality of frailty index and vestibular disorders.

Vestibular disorders are mainly characterized by dysfunction of the vestibular system, involving body posture and motion perception, eye movement control, posture, gait, and spatial positioning. According to the proposed structure of the international classification of vestibular disorders (ICVD), there are four layers about vestibular disorders: symptoms and signs; clinical syndromes; diseases/disorders; and pathophysiologic mechanisms. Four categories of vestibular symptoms are described by the ICVD: vertigo, dizziness, vestibulovisual symptoms, and postural symptoms (Bisdorff et al., 2015). Vertigo and dizziness are one of the most common complaints in patients with vestibular disorders. In a previous study, researchers have found that patients with intractable dizziness are more likely to develop frailty (Gomez et al., 2011). According to research, community-dwelling elderly adults with frailty are much more likely to experience dizziness. Furthermore, Researchers have discovered that older adults who report dizziness tend to be physically frail, have more chronic diseases and sensory impairments (de Moraes et al., 2011, 2013; Gomez et al., 2011; Kammerlind et al., 2016). Dizziness, unsteadiness, or lightheadedness is associated with frailty, and in fully adjusted models, frailty was still related to dizziness, unsteadiness, or lightheadedness (O'Connell et al., 2015; Goshtasbi et al., 2020). Because of the limitations of observational epidemiological studies in eliminating bias (for example, reverse causation and confounding factors), while observational studies have reported a relationship between frailty and vestibular disease, little is known about their causal relationship.

The results of our study suggested that vestibular disorders may be independently affected by the genetic liability to frailty index. Notably, Atkins et al. (2021) demonstrated that multiple traits including BMI, CRP, IBD, and smoking initiation are associated with the risk of frailty (Liu et al., 2022). In this study, multivariable MR analysis including the four traits was undertaken in order to evaluate the causal relationship between genetically predicted frailty index and the risk of vestibular disorders. The results indicate that frailty index may be still associated with an increased risk of vestibular disorders after adjustment for BMI, CRP, IBD and smoking. According to the results of the systematic review and meta-analysis conducted by Yuan et al. (2021) obesity or underweight is associated with an increased risk of frailty in community-dwelling older adults. Studies also found that frailty are associated with CRP and IBD (Soysal et al., 2016; Kochar et al., 2021). Smoking is one of the main causes of health problems worldwide and can also lead to an increased risk of frailty. In addition, smoking-associated frailty may be linked to epigenetic changes (Gao et al., 2017). Notably, it is necessary to conduct further research to replicate our findings in relation to frailty and vestibular disorders due to the potential confounders.

The mechanisms involved in frailty resulting in vestibular disease are complex and poorly understood. Frailty is a complicated and prevalent age-related clinical syndrome characterized by a decline in physiological capacities across multiple organs or (and) systems (Vermeiren et al., 2016; Cesari et al., 2017; Dent et al., 2019). There is a close connection between aging and frailty (Mitnitski et al., 2017). A number of studies have demonstrated that vestibular function declines with aging (Brosel et al., 2016). It is believed that the central vestibular system, vision, and proprioception slowly deteriorate with aging, contributing to vestibular compensation mechanisms degrading. The vestibular system is revealed to lose neural cells with aging through anatomical studies (Bouccara et al., 2018; Krager, 2018; Vanspauwen, 2018). It is becoming increasingly clear that biological aging (vs. chronological aging) contributes to the development of chronic diseases and physical frailty at the molecular and cellular level which can lead to decline and death (Fougere et al., 2017). Furthermore, a cross-sectional study has shown that frailty is independently associated with mortality and prolonged hospital stays following vestibular schwannoma resection (Dicpinigaitis et al., 2021; Tang et al., 2022). Compared with advanced patient age alone, frailty may be more accurate for predicting vestibular schwannoma resection outcomes. Health-related outcomes are more likely to be determined by frailty than by age, and targeted interventions may prevent or mitigate frailty (de Labra et al., 2015; Wilson et al., 2015; Bray et al., 2016). Frailty starts before age 65 in many studies, and not all older people develop frailty, despite their advanced age (Dent et al., 2019). Most intervention trials involve older people, despite the fact that frailty can affect people at any age (especially if comorbid conditions are present). Frailty index is an important indicator of accelerated biological aging (when the organism exceeds its actual age). Research has proved the frailty index score was a remarkable predictor of morbidity and mortality in chronologically young orthopedic trauma patients (Kojima et al., 2018; Grabovac et al., 2019). Notably, the prevalence of frailty and prefrailty was 45.9% in the young adults in a previous study (Yasuda, 2021). A growing number of young people suffer from benign paroxysmal positional vertigo (BPPV, one of the most primary vestibular disorders), in which frailty is one of the risk factors for BPPV in young people (Wang et al., 2021). Therefore, in addition to vestibular degeneration associated with frailty, more mechanisms need to be explored as frailty becomes younger.

There are several strengths to the study. It is the first report using summary level data from large GWASs to confirm the potential causal relationship between frailty index and vestibular disorders. In order to verify the hypothesis, several sensitivity analyses were carried out. Additionally, multivariate analysis was performed in order to adjust for confounding factors. And to a certain extent make our results more reliable. However, there are several limitations in our MR analysis. First, given the classification of the original data, we could not further subdivide the pressure type of vestibular disorders, and thus we could only analyze the vestibular disorders as a whole. Second, although Mendelian randomization has been shown to be a powerful method to assess the causality between frailty index and vestibular disorders, the two-sample MR analysis only provides an estimate of the putative causal effect, and further studies are required to estimate a direct causal effect of frailty on vestibular disorders. Third, a reverse causal association (effect of vestibular disorders on frailty) was not evaluated in this study. The fourth limitation is that the GWAS data were compiled for individuals of European descent, which means that the population at large might not be fully represented by our results. Fifth, the two GWAS datasets are from European populations, there will be an overlap of samples. Over-fitting and instrument bias become more pronounced as overlap between samples increases, similar to those observed in one-sample MR. Last, the two European samples could differ substantially according to population characteristics such as socio-economic background, which also could affect the interpretation of causal estimates.

## Conclusion

In our study, we found that vestibular disorders may be causally related to frailty index. Notably, considering the limitations of this study, the causal effects between frailty index and vestibular disorders need further investigation. These results support the importance of effectively managing frailty which may minimize vestibular disorders and improve the quality of life for those with vestibular disorders.

## Data availability statement

The original contributions presented in the study are included in the article/Supplementary material, further inquiries can be directed to the corresponding author/s.

## Author contributions

GX and CQ conceptualized and designed the study. GX, HW, and JH prepared and analyzed the data and drafted the manuscript. LL, TZ, MZ, and XL contributed to interpretation and editing of the manuscript. All authors contributed to the article and approved the submitted version.

## Funding

This work was supported by the National Natural Science Foundation of China (Grant Number 72074225), the Philosophy and Social Science Foundation of Hunan Province (Grant Number 19YBA351), and the Key R&D Plan of Hunan Province (Grant Number 2020SK2089). The funding sources played no role in conducting the research and preparing the article.

## Conflict of interest

The authors declare that the research was conducted in the absence of any commercial or financial relationships that could be construed as a potential conflict of interest.

## Publisher's note

All claims expressed in this article are solely those of the authors and do not necessarily represent those of their affiliated organizations, or those of the publisher, the editors and the reviewers. Any product that may be evaluated in this article, or claim that may be made by its manufacturer, is not guaranteed or endorsed by the publisher.
