# Supplementary material for: Estimating the causal effect of frailty index on vestibular disorders: A two-sample Mendelian randomization
Source: Front Neurosci. 2022 Aug 24;16:990682. doi: 10.3389/fnins.2022.990682 (PMC9448900; doi:10.3389/fnins.2022.990682)

Supplementary Material

***F*-statistic**

We computed the *F*-statistic of each SNP using the following formula ^[1,2]^: *F*-statistic = (β/SE)^2^, where Beta is the per allele effect size of the association between each SNP and phenotype, SE is the standard error.

**References:**

1. Chen L, Yang H, Li H, He C, Yang L, Lv G. Insights into modifiable risk factors of cholelithiasis: A Mendelian randomization study [published online ahead of print, 2021 Oct 8]. Hepatology. 2021;10.1002/hep.32183.

2. Jiang J, Shao M, Wu X. Vitamin D and risk of ankylosing spondylitis: A two-sample mendelian randomization study. Hum Immunol. 2022;83(1):81-85.

**Supplemental tables**

**Table S1.** Details of datasets used for analyses.

| **First author** | **Year** | **Exposure/Outcome** | **Participants** | **Numbers of variants** | **Web source if publicly available** |
| --- | --- | --- | --- | --- | --- |
| Atkins JL | 2021 | Frailty index | 175,226 individuals of European ancestry | 758971 | https://gwas.mrcieu.ac.uk/datasets/ebi-a-GCST90020053/ |
| Ben Elsworth | 2018 | vestibular disorders | 462,933 individuals (4,012 cases and 458,921 controls) of European ancestry | 9851867 | https://gwas.mrcieu.ac.uk/datasets/ukb-b-5188/ |

**Table S2.** Questionnaire items from the baseline UK Biobank assessment used to compose the frailty index

| Type of  deficit | Item | Trait | Categories | Coding in FI item | Score of 1 n (%)† |
| --- | --- | --- | --- | --- | --- |
| *Sensory* | 1 | Glaucoma *  Cataracts *  Hearing difficulty | no,yes  no,yes  no, yes, completely deaf | Categorised 0/1  Categorised 0/1  Categorised 0/1 (combined yes/deaf groups as 1) | 3,728 (2.26)  8,993 (5.46)  52,506 (31.90) |
| *Cranial* | 2 | Migraine *  Dental problems | no,yes  ulcers, painful gums, bleeding gums, loose teeth, toothache, dentures | Categorised 0/1  Categorised 0/1 for none vs. any | 3,863 (2.35)  71,494 (43.43) |
| *Mental wellbeing* | 3 | Self-rated health  Fatigue: frequency of tiredness / lethargy in last two weeks  Sleep: experience of sleeplessness/insomnia  Depressed feelings: frequency in last two weeks  Self-described nervous personality  Severe anxiety/ panic attacks *  Common to feel loneliness  Sense of misery (ever/never) | excellent, good, fair, poor  not at all, several days, more than half, nearly  never/rarely, sometimes, usually  not at all, several days, more than half, nearly every day  no, yes  no, yes  no, yes  no, yes | 0 – excellent; 0.25 – good; 0.5 - fair; 1 – poor  0, 0.25, 0.5, 1, respectively  Categorised 0, 0.5, 1, respectively  0 – not at all, 0.5 – several days, 0.75 - more than half, 1 – nearly every day  Categorised 0/1  Categorised 0/1  Categorised 0/1  Categorised 0/1 | 6,231 (3.79)  6,928 (4.21)  49,304 (29.95)  1,834 (1.11)  35,478 (21.55)  1,936 (1.18)  24,005 (14.58)  57,738 (35.08) |
| *Infirmity* | 4 | Infirmity: long-standing illness or disability  Falls in last year  Fractures/broken bones in last five years | no, yes  categorical: no falls, one fall, more than one  no, yes | Categorised 0/1  0, 0.5, 1, respectively  Categorised 0/1 | 60,831 (36.95)  10,831 (6.58)  15,881 (9.65) |
| *Cardiometabolic* | 5 | Diabetes *  Myocardial infarction *  Angina *  Stroke *  High blood pressure *  Hypothyroidism *  Deep-vein thrombosis *  High cholesterol * | no, yes  no, yes  no, yes  no, yes  no, yes  no, yes  no, yes  no, yes | Categorised 0/1  Categorised 0/1  Categorised 0/1  Categorised 0/1  Categorised 0/1  Categorised 0/1  Categorised 0/1  Categorised 0/1 | 10,881 (6.61)  6,221 (3.78)  8,779 (5.33)  3,756 (2.28)  59,258 (36.00)  9,586 (5.82)  4,450 (2.70)  45,052 (27.37) |
| Respiratory | 6 | Breathing: wheeze in last year  Pneumonia *  Chronic bronchitis/emphysema *  Asthma * | no, yes  no, yes  no, yes  no, yes | Categorised 0/1  Categorised 0/1  Categorised 0/1  Categorised 0/1 | 34,566 (21.00)  2,800 (1.70)  3,839 (2.33)  17,447 (10.60) |
| *Musculoskeletal* | 7 | Rheumatoid arthritis *  Osteoarthritis *  Gout *  Osteoporosis * | no, yes  no, yes  no, yes  no, yes | Categorised 0/1  Categorised 0/1  Categorised 0/1  Categorised 0/1 | 2,314 (1.41)  19,804 (12.03)  3,208 (1.95)  4,187 (2.54) |
| *Immunological* | 8 | Hayfever, allergic rhinitis or eczema *  Psoriasis * | no, yes  no, yes | Categorised 0/1  Categorised 0/1 | 32,727 (19.88)  1,763 (1.07) |
| *Cancer* | 9 | Any cancer diagnosis *  Multiple cancers diagnosed (number reported) | no, yes  Range from 0 to 6 | Categorised 0/1  0 - no cancer or single cancer, 1 - multiple cancers | 19,068 (11.58)  1,430 (0.87) |
| *Pain* | 10 | Chest pain  Head and/or neck pain  Back pain  Stomach/abdominal pain  Hip pain  Knee pain  Whole-body pain  Facial pain  Sciatica * | no, yes  no, yes (combining responses to pain in head and neck/shoulders)  no, yes  no, yes  no, yes  no, yes  no, yes  no, yes  no, yes | Categorised 0/1  Categorised 0/1  Categorised 0/1  Categorised 0/1  Categorised 0/1  Categorised 0/1  Categorised 0/1  Categorised 0/1  Categorised 0/1 | 25,041 (15.21)  49,029 (29.78)  40,764 (24.76)  10,703 (6.50)  21,192 (12.87)  37,661 (22.88)  2,468 (1.50)  2,402 (1.46)  1,643 (1.00) |
| *Gastrointestinal* | 11 | Gastric reflux *  Hiatus hernia *  Gall stones *  Diverticulitis * | no, yes  no, yes  no, yes  no, yes | Categorised 0/1  Categorised 0/1  Categorised 0/1  Categorised 0/1 | 8,371 (5.09)  5,076 (3.08)  3,593 (2.18)  2,748 (1.67) |
| FI score as previously validated in UK Biobank by Williams et al28.  †N=164,610 (60-70 year olds; European descent; complete case analysis of all 49 FI components).  * Participants reported medically diagnosed conditions for these items. | | | | | |

**Table S3.** Questionnaire items from TwinGene used to compose the Frailty Index

| No. | Questions | Coding |
| --- | --- | --- |
| 1 | How do you estimate your general health? | Excellent=0, Good=0.25, Average=0.5, Not so good=0.75, Bad=1 |
| 2 | Do you think your health status prevents you from doing things you want to do? | Not at all=0, To some extent=0.5, A great deal=0 |
| 3 | How many times a year do you get serious infections (other than respiratory)? | 0-1 times=0, 2-4 times=0.5, 5 times or more =1 |
| 4 | Do you have buzzing in the ears? | Both ears or one ear=1, No=0 |
| 5 | Do you have or have you had angina pectoris | No=0, Yes=1 |
| 6 | Do you have or have you had heart attack | No=0, Yes=1 |
| 7 | Do you have or have you had heart failure | No=0, Yes=1 |
| 8 | Do you have or have you had high blood pressure | No=0, Yes=1 |
| 9 | Do you have or have you had lipid disorder, for example high cholesterol or high triglycerides | No=0, Yes=1 |
| 10 | Do you have or have you had vascular spasm in the legs (intermittent claudication) | No=0, Yes=1 |
| 11 | Do you have or have you had clot in the leg (venous thrombosis) | No=0, Yes=1 |
| 12 | Do you have or have you had cerebral hemorrhage or clot in the brain (stroke) | No=0, Yes=1 |
| 13 | Do you have or have you had TIA attacks (temporary weakness or paralysis or reduction of sensibility) | No=0, Yes=1 |
| 14 | Do you have or have you had irregular cardiac rhythm/atrial fibrillation | No=0, Yes=1 |
| 15 | Do you have or have you had chronic lung disease (including chronic bronchitis and emphysema) | No=0, Yes=1 |
| 16 | Do you have or have you had dizziness | No=0, Yes=1 |
| 17 | Do you have or have you had rheumatoid arthritis | No=0, Yes=1 |
| 18 | Do you have or have you had knee joint problem | No=0, Yes=1 |
| 19 | Do you have or have you had sciatica | No=0, Yes=1 |
| 20 | Do you have or have you had osteoporosis | No=0, Yes=1 |
| 21 | Do you have or have you had hip joint problem | No=0, Yes=1 |
| 22 | Do you have or have you had back pain | No=0, Yes=1 |
| 23 | Do you have or have you had neck pain | No=0, Yes=1 |
| 24 | Do you have or have you had diabetes (including old age diabetes, and excluding pregnancy diabetes) | No=0, Yes=1 |
| 25 | Do you have or have you had goiter | No=0, Yes=1 |
| 26 | Do you have or have you had glandular diseases (excluding goiter) | No=0, Yes=1 |
| 27 | Do you have or have you had gall bladder problem | No=0, Yes=1 |
| 28 | Do you have or have you had liver disease (for example, cirrhosis) | No=0, Yes=1 |
| 29 | Do you have or have you had gout | No=0, Yes=1 |
| 30 | Do you have or have you had kidney disease | No=0, Yes=1 |
| 31 | Do you have or have you had stomach or intestine problems | No=0, Yes=1 |
| 32 | Do you have or have you had recurring urinary tract problems | No=0, Yes=1 |
| 33 | Do you have or have you had cancer, tumor disease or leukemia | No=0, Yes=1 |
| 34 | Do you have or have you had migraine | No=0, Yes=1 |
| 35 | Do you have or have you had asthma | No=0, Yes=1 |
| 36 | Do you have or have you had allergy | No=0, Yes=1 |
| 37 | Do you have recurrent periods of coughing? | No=0, Yes=1 |
| 38 | You felt depressed. Never, seldom, often or always during the past week? | Never or almost never=0, Seldom=0.5, Often, always or almost always=1 |
| 39 | You were happy. Never, seldom, often or always during the past week? | Never or almost never=1, Seldom=0.5, Often, always or almost always=0 |
| 40 | You felt lonely. Never, seldom, often or always during the past week? | Never or almost never=0, Seldom=0.5, Often, always or almost always=1 |
| 41 | Do you have or have you had any physical handicap | No=0, Yes=1 |
| 42 | Do you have or have you had Crohn's disease or Ulcerative colitis | No=0, Yes=1 |
| 43 | How is your vision? | Good=0, Reduced=0.5, Highly reduced or blind=1 |
| 44 | How is your hearing? | Good=0, Reduced=0.5, Highly reduced=1 |

| SNP | b | se | *p* |
| --- | --- | --- | --- |
| rs10891490 | 0.006748725 | 0.002432376 | 0.0060^**^ |
| rs12739243 | 0.008894798 | 0.002443438 | 0.0003^**^ |
| rs1363103 | 0.007984407 | 0.002432587 | 0.0016^*^ |
| rs17612102 | 0.007741118 | 0.002431457 | 0.0016^*^ |
| rs2071207 | 0.007474499 | 0.002434063 | 0.0026^*^ |
| rs2396766 | 0.007194827 | 0.002444381 | 0.0036^*^ |
| rs374943348 | 0.007818563 | 0.002472049 | 0.0016^*^ |
| rs3959554 | 0.007481303 | 0.002433623 | 0.0026^*^ |
| rs4146140 | 0.007869403 | 0.002437193 | 0.0016^*^ |
| rs4952693 | 0.007826727 | 0.002434265 | 0.0016^*^ |
| rs56299474 | 0.008026393 | 0.002431034 | 0.0010^*^ |
| rs583514 | 0.007369885 | 0.002442932 | 0.0026 |
| rs8089807 | 0.006917216 | 0.002438236 | 0.0046 |
| rs82334 | 0.006933089 | 0.002449602 | 0.0047 |
| rs9275160 | 0.006292711 | 0.002646611 | 0.0175 |
| All | 0.007517079 | 0.0023689 | 0.0016 |

**Table S4.** Detailed results of leave-one-out plot.

| **Exposure** | **covariates** | **Outcome** | **Beta** | **SE** | **OR** | **LCI** | **UCI** | ***p*-val** | **FDR *p*--val** | **SNPs** |
| --- | --- | --- | --- | --- | --- | --- | --- | --- | --- | --- |
| ebi-a-GCST90020053 (Frailty index) | ieu-a-2 (Body mass index) | ukb-b-5188 | 0.0061243088 | 0.0029056321 | 1.006 | 1.000 | 1.012 | 0.035^*^ | 0.035^*^ | 8 |
| ebi-a-GCST90020053 (Frailty index) | ebi-a-GCST008055 (C-reactive protein) | ukb-b-5188 | 7.498041e-03 | 0.0020901074 | 1.008 | 1.003 | 1.012 | 0.0003^**^ | 0.0007^**^ | 13 |
| ebi-a-GCST90020053 (Frailty index) | ieu-a-31 (Inflammatory bowel disease) | ukb-b-5188 | 6.220261e-03 | 0.002793623 | 1.006 | 1.001 | 1.012 | 0.026^*^ | 0.035^*^ | 11 |
| ebi-a-GCST90020053 (Frailty index) | ieu-b-4877 smoking initiation | ukb-b-5188 | 0.0079049138 | 0.002186002 | 1.008 | 1.004 | 1.012 | 0.0003^**^ | 0.0007^**^ | 10 |

**Table S5.** Multivariable Mendelian randomization analysis of the association between genetically predicted FI and risk of vestibular disorder

**Table S6** | Association of associated genetypes with vestibular disorders using various methods.

| **Exposure** | **Outcome** | *p*-Fixed-effect IVW | *p*-Weighted median | *p*-Simple mode | *p-*Weighted mode | *p*-MR-Egger |
| --- | --- | --- | --- | --- | --- | --- |
| ieu-a-2  (Body mass index) | ukb-b-5188  （vestibular disorders） | 0.08 | 0.09 | 0.5 | 0.24 | 0.58 |
| ebi-a-GCST008055  (C-reactive protein) | ukb-b-5188  （vestibular disorders） | 0.89 | 0.34 | 0.92 | 0.42 | 0.3 |
| ieu-a-31  (Inflammatory bowel disease) | ukb-b-5188  （vestibular disorders） | 0.91 | 0.07 | 0.29 | 0.18 | 0.49 |
| ieu-b-4877  smoking initiation | ukb-b-5188  （vestibular disorders） | 0.50 | 0.61 | 0.99 | 0.08 | 0.21 |

**p < 0.01；*p < 0.05；OR, odds ratio; LCI, lower confidence interval; UCI, upper; IVW, inverse-variance weighted.

**Supplemental figure**

**Figure S1.** Funnel plot of the MR analysis for causal association of frailty index with vestibular disorders. X axis presented the causal estimates and Y axis presented the inverse SE. The dots indicated each SNP, and the line indicated the overall estimate using fixed-effect IVW method. *MR*, Mendelian randomization; *SE*, standard error; *SNP*, single nucleotide polymorphism; *IVW*, inverse variance-weighted.


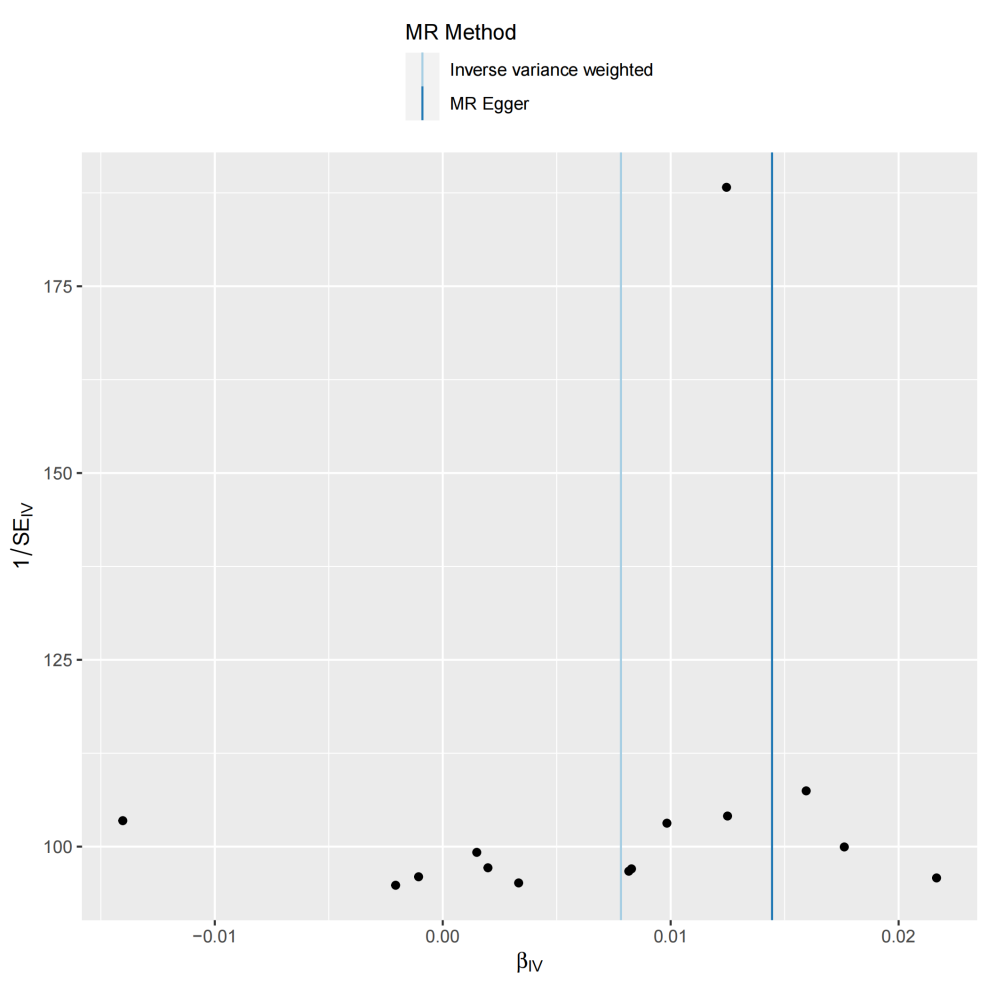

Supplement: Supplementary file 1 [file Data_Sheet_1.docx]
